# Tendon Transfers, Nerve Grafts, and Nerve Transfers for Isolated Radial Nerve Palsy: A Systematic Review and Analysis

**DOI:** 10.1177/15589447221150516

**Published:** 2023-01-24

**Authors:** Nirbhay S. Jain, Meaghan L. Barr, Daniel Kim, Neil F. Jones

**Affiliations:** 1 University of California, Los Angeles, USA

**Keywords:** radial nerve palsy, systematic review, pooled analysis, nerve transfer, tendon transfer, nerve graft

## Abstract

**Background:**

Isolated radial nerve palsy is a debilitating injury that may potentially be reconstructed with either tendon transfers, nerve grafts, or nerve transfers. Currently, there is no consensus on the optimal technique for reconstruction. We performed a systematic review and analysis to determine which surgical intervention provides the best clinical outcomes.

**Methods:**

A systematic review was conducted according to PRISMA guidelines. Twenty-nine papers met inclusion criteria. Grading scales of function and strength were converted into a tripartite scoring system to compare outcomes between techniques. χ^2^ analyses were performed with a *P* value < .05.

**Results:**

Seven hundred fifty-four patients were analyzed. Tendon transfers resulted in the highest percentage of good outcomes (82%) and the lowest percentage of poor outcomes (9%). Tendon transfers were superior to nerve grafts and nerve transfers for restoration of wrist extension. Nerve transfers for wrist extension were superior to nerve transfers for finger extension. Nerve grafts and nerve transfers had equivalent rates of good and poor clinical outcomes.

**Conclusions:**

This study analyzed reported outcomes of tendon transfers, nerve grafts, and nerve transfers for reconstruction of isolated radial nerve palsy. On pooled analysis, tendon transfers had higher rates of superior clinical outcomes as compared with nerve transfers and nerve grafts. Tendon transfers should be considered first-line reconstruction for isolated radial nerve palsy as nerve-based reconstruction is less predictable and reproducible.

## Introduction

Isolated radial nerve palsy results in loss of wrist, finger, and thumb extension.^1^ These deficits limit activities of daily living and decrease quality of life. Radial nerve deficits are frequently secondary to trauma; up to 20% of humerus fractures are complicated by radial nerve palsy. Other causes include iatrogenic injury, birth injury, and oncologic resections.

Given the significant morbidity of radial nerve palsy, surgical procedures have been developed to treat affected patients. The classic intervention is tendon transfer, in which an expendable donor muscle is used to perform the absent function. Although tendon transfer provides gross function, some feel outcomes lack subtle motor control.^2^

Although nerve grafting restores function without donor muscle sacrifice, it requires a secondary donor nerve and accessible proximal and distal nerve stumps.^3^ As with any nerve-based reconstruction, reinnervation of the target muscle must be performed within 12 to 18 months from the date of injury, or irreversible motor end plate atrophy guarantees reconstructive failure.^4^ The best outcomes occur in young patients with small nerve gaps repaired 3 to 6 months after injury.

More recently, nerve transfers have become popular. These procedures combine the principles of tendon transfers and nerve grafting by coapting a terminal motor nerve from an expendable muscle to the motor nerve of the paralyzed target muscle. Although postoperative motor control may be more nuanced, the operation is dependent on nerve fiber growth across a nerve coaptation, requires lengthy rehabilitation periods, and has less reliable results.^2^

There is no consensus on the optimal reconstructive technique for isolated radial nerve palsy. We performed a systematic review and pooled analysis to determine which technique was superior, as determined by the proportion of patients who achieved good results as measured by grading rubrics of function and strength. We then assessed comparisons based on the anatomical level of function, namely, assessing outcomes of each technique at the wrist and finger separately.

## Methods

### Literature Search

A systematic review was performed according to the Preferred Reporting for Systematic Reviews and Meta-analyses (PRISMA) statement guidelines. The search was performed using PubMed/MEDLINE, EMBASE, ScienceDirect, Cochrane Library with the Boolean search string: (radial nerve) AND (palsy OR injury OR paralysis OR lesion) AND (tendon OR nerve) AND (graft OR transfer OR reconstruction OR reinnervation). The Boolean string was adapted for each database according to their specific conditions. Inclusion and exclusion criteria were determined before review was conducted. Screening criteria excluded nonjournal articles, nonsurgical studies, and articles written in languages other than English without translation. Full-text review excluded any articles without radial nerve palsy or outcomes reporting. Only patients with complete radial nerve palsy (Sunderland grade V) were included. The process of article selection was repeated by 2 independent reviewers to ensure transparency and consistency, and the senior author served as judge. Any disagreement was resolved by a third reviewer. The search was performed again toward the end of the review to ensure consistent results from the search string and selection criteria.

### Data Extraction

Relevant patients were extracted from selected papers. Inclusion criteria were patients who underwent tendon transfer, nerve transfer, or nerve grafting for isolated complete radial nerve palsy, regardless of specific donors. For patients in articles with multiple types of injuries or differing techniques, only patients who met inclusion qualifications were included for further analysis.

Outcomes were classified as good response, neutral response, or poor response based on the scale used by the authors in each paper. Internal scales were given 3 scores and were translated into a uniform scale as appropriate. The grading scale conversion is displayed in Table 1.^5-9^

**Table 1.** Equivalences of Grading Scales Used.

Grade	MRC	Bincaz	LSUHS	Haas	Zachary
Good	4-5	>6	>3	Antigravity	Very good/good
Neutral	3	—	3	Gravity	Satisfactory
Poor	0-2	<6	<3	Unable to overcome gravity	Bad

*Note.* MRC = Medical Research Council; LSUHS = Louisiana State University Health System.

Several papers included functional outcomes at different anatomical levels, namely, at the wrist and finger joints. For these patients, the overall grade assigned was the highest level of strength achieved, but subgrades were noted at each joint for subgroup analysis.

For patients undergoing tendon transfer or nerve transfer, subgroup extraction based on individual donor/recipient muscle pairs was not performed given heterogeneity in patient populations and inability to control for outcomes of individual transfers.

### Statistical Analysis

Grouped analyses were performed to determine superiority of one technique over the others. A χ^2^ test with Bonferroni corrections was run with a significance level of *P* < .05 for the overall grade for each patient per technique. These comparisons were also run between techniques for each functional level of recovery. Anatomical comparisons and grading scale comparisons were conducted within each technique to determine whether the degree of recovery changed depending on the function measured or how measurements were taken. Data analyses were done in SPSS (IBM, Armonk, NY). Meta-analyses were considered but not performed; individual papers did not provide statistical analysis on which a meta-analysis could be performed.

## Results

### Review Results

Literature search produced 2735 articles, with 1989 screened, 82 reviewed, and 29 included. These were published between 1946 and 2021 and included a global scope (Table 2).^9-38^

**Table 2.** Papers Analyzed.

Author	Journal	Year	Country	Surgery	Transfers	N	Sex	Age (year)	Injury	Preoperative interval	Last visit	Distance subgroups	Good	Neutral	Poor	Classification system
Bertelli et al^13^	*J Neurosurg*	2021	Brazil	Nerve transfer	PT → ECRB	5	3 M	30	Trauma	NA	24 mo	Yes	5	0	0	MRC
Bertelli^14^	*JHS*	2020	Brazil	Nerve transfer	PQ → ECRB, FCR → PIN	14	13 M	28	NA	NA	26 mo	Yes	10	4	0	MRC
Emamhadi and Andalib^15^	*World Neurosurg*	2018	Iran	Nerve transfer	Ulnar → triceps, FDS/FCR → PIN/ECRB	1	1 M	21	Trauma	6 mo	12 mo	Yes	1	0	0	MRC
Emamhadi et al^16^	*Acta Neurochir*	2020	Iran	Nerve transfer	Ulnar → triceps, FDS/FCR → PIN/ECRB	2	2 M	30	Trauma	12 mo	7 mo	Yes	2	0	0	MRC
Garcia-Lopez et al^17^	*JHS*	2014	Spain	Nerve transfer	FCR → PIN, PT → ECRL	6	5 M	25	Trauma	5 mo	20 mo	Yes	4	2	0	MRC
Lowe et al^18^	*PRS*	2002	USA	Nerve transfer	FDS → PIN	1	M	51	Trauma	6 mo	14 mo	Yes	1	0	0	MRC
Rasulić et al^11^	*World Neurosurg*	2017	Serbia	Nerve transfer	FDS → ECRB, FCR → PIN	4	NA	NA	NA	NA	NA	No	4	0	0	Internal
Ray and Mackinnon^19^	*J Hand Surg Am*	2011	USA	Nerve transfer	FDS/FCR → PIN/ECRB	10	NA	48	NA	5.7 mo	20.3 mo	Yes	6	1	3	MRC
Sallam et al^20^	*HSSJ*	2016	Egypt	Nerve transfer	PIN → ECRB, FCR → PL, FDS → PIN	21	NA	NA	NA	NA	NA	Yes	5	11	5	MRC
Agarwal et al^21^	*J Clin Orthop Trauma*	2020	India	Tendon transfer	PT → ECRL/ECRB, FCR → EDC, PL → EPL	53	47 M	35	Trauma	9 mo	9 mo	No	50	0	3	Bincaz
Agrawal and Gupta^38^	*J Clin Diag Res*	2021	India	Tendon transfer	PT → ECRL/ECRB, FCU → EDC	26	NA	38	Trauma	3 mo	NA	No	24	0	2	Bincaz
Al-Qattan^22^	*Annals*	2013	Saudi Arabia	Tendon transfer	FCU → EDC/EIP/EPL	4	4 M	32	Trauma	NA	7 mo	No	2	0	2	Bincaz
Al-Qattan^37^	*J Hand Surg Eur*	2012	Saudi Arabia	Tendon transfer	PT → ECRL/ECRB, FCU → EDC	15	10 M	28	Trauma	NA	6 mo	Yes	12	0	3	Bincaz
Bertelli^14^	*JHS*	2020	Brazil	Tendon transfer	PT → ECRL/ECRB, FCU → EDC, PL → EPL	13	12 M	30	NA	NA	18 mo	Yes	11	2	0	MRC
Bevin^30^	*Hand*	1976	USA	Tendon transfer	PT → ECRL/ECRB, FCU → EDC, PL → EPL	21	NA	NA	NA	NA	NA	No	21	0	0	Zachary
Carls et al^23^	*Z Ortho Ihre Grenzgeb*	2001	Germany	Tendon transfer	FCU → EDC, PL → EPL	10	NA	NA	NA	NA	NA	No	9	1	0	Haas
Chuinard et al^32^	*JHS*	1978	USA	Tendon transfer	PT → ECRL/ECRB, FDS → EDC, PL → EPL	19	15 M	8-60	Trauma (15)	1-20 y	3-6 y	Yes	19	0	0	Zachary
Kruft et al^24^	*PRS*	1997	Germany	Tendon transfer	PT → ECRL/ECRB	43	36 M	35	Trauma	22 mo	Up to 12 y	No	31	12	0	Zachary
Moussavi et al^25^	*Ind J Orthop*	2011	Iran	Tendon transfer	PT → ECRL/ECRB, FCU → EDC	18	14 M	26	NA	NA	NA	Yes	15	0	3	Bincaz
Moussavi et al^25^	*Ind J Orthop*	2011	Iran	Tendon transfer	PT → ECRL/ECRB, FCR → EDC	10	9 M	27	NA	NA	NA	Yes	8	0	2	Bincaz
Moussavi et al^25^	*Ind J Orthop*	2011	Iran	Tendon transfer	PT → ECRL/ECRB, FDS → EDC	13	10 M	27	NA	NA	NA	Yes	11	0	2	Bincaz
Raskin and Wilgis^29^	*JHS*	1995	USA	Tendon transfer	PT → ECRL/ECRB, FCU → EDC, PL → EPL	6	NA	NA	NA	NA	NA	Yes	6	0	0	Zachary
Ropars et al^26^	*J Hand Surg Br*	2006	France	Tendon transfer	PT → ECRL/ECRB, FCU → EDC, ECRL → ECU/ECRB, PL → EPL	15	3 M	38.5	Trauma (12), excision (3)	27 mo	10 y	No	13	0	2	Bincaz
Thomsen and Rasmussen^31^	*Scand J Plast Recon*	1969	Denmark	Tendon transfer	PT → ECRL/ECRB, FCU → EDC, PL → EPL	5	4 M	31.6	Trauma	NA	5.6 y	Yes	4	1	0	Zachary
Kumar Vyas et al^27^	*J Clin Orthop Trauma*	2020	India	Tendon transfer	PT → ECRL/ECRB, FDS → EDC, PL → EPL	15	14 M	<50	NA	NA	NA	No	10	0	5	Bincaz
Zachary^36^	*Br J Surg*	1946	UK	Tendon transfer	PT → ECRL/ECRB, FCU → EDC	53	NA	NA	Trauma	NA	NA	Yes	31	14	8	Zachary
Bertelli and Ghizoni^10^	*J Neurosurg*	2016	Brazil	Nerve graft	—	13	13 M	26	Trauma	6 mo	36 mo	Yes	3	2	8	MRC
Dolenc^28^	*Acta Neurochir*	1977	Slovenia	Nerve graft	—	14	NA	NA	NA	NA	NA	No	11	3	0	MRC
Rasulić et al^11^	*World Neurosurg*	2017	Serbia	Nerve graft	—	7	NA	NA	NA	NA	NA	No	5	0	2	Internal
Roganovic et al^12^	*Acta Neurochir*	2007	Serbia	Nerve graft	—	23	NA	33.5	NA	3.3 mo	NA	No	19	2	2	MRC
Schwaiger et al^9^	*J Clin Med*	2020	Austria	Nerve graft	—	4	NA	31	Trauma (3), neuroma (1)	5.5 mo	3.3 y	No	2	2	0	LSUHS
Seddon^34^	*Br J Surg*	1947	UK	Nerve graft	—	3	NA	NA	NA	NA	5 y	No	1	2	0	MRC
Seddon^33^	*Proc R Soc Med*	1949	UK	Nerve graft	—	45	NA	NA	NA	NA	5 y	No	0	22	23	MRC
Shergill et al^35^	*J Bone Joint Surg Br*	2001	UK	Nerve graft		242	NA	28	NA	NA	NA	No	72	68	102	MRC

*Note.* PT = pronator teres; ECRB = extensor carpi radialis brevis; ECRL = extensor carpi radialis longus; FCU = flexor carpi ulnaris; FCR = flexor carpi radialis; FDS = flexor digitorum superficialis; PL = palmaris longus; EPL = extensor pollicis longus; EDC = extensor digitorum communis; PQ = pronator quadratus; PIN = posterior interosseous nerve; MRC = Medical Research Council; LSUHS = Louisiana State University Health System; NA = not available; *JHS* = Journal of Hand Surgery; PRS = *Plastic and Reconstructive Surgery.*

### Pooled Analysis Patient Summary

In total, 754 patients were included: 339 for tendon transfer, 351 for nerve grafting, and 64 for nerve transfer.

Common tendon transfers included pronator teres (PT) to the extensor carpi radialis brevis (ECRB) and longus (ECRL); the flexor carpi radialis (FCR), flexor digitorum superficialis (FDS), or flexor carpi ulnaris (FCU) to the extensor digitorum communis (EDC); and the palmaris longus (PL) to the extensor pollicis longus (EPL). Of these, 428 patients (57%) had good, 154 patients (20%) had neutral, and 177 patients (24%) had poor results. Of the 10 patients with FCR-based reconstruction, 80% had good results. For the FCU-based reconstruction 78% had good results (n = 160). For FDS-based reconstruction, 79% had good results (n = 90).

All nerve transfers had an element of FDS/FCR to posterior interosseous nerve (PIN)/ECRB, most typically FDS to ECRB and FCR to PIN, although this varied in some case reports.

In total, 171 patients (23%) had results described at the wrist and 226 patients (30%) had results described at the fingers. Not all patients had outcomes at different levels. Age ranged from 20 to 50 years old. More than 500 patients presented after trauma. Time from injury to surgery varied, with tendon transfers occurring 9 to 27 months after injury and nerve reconstruction occurring 3 to 12 months after injury. Follow-up averaged 1 year after surgery, although several patients were seen a decade later. A total of 413 patients (55%) were evaluated by Medical Research Council (MRC) grade, 169 patients (23%) by the Bincaz scale, 147 patients (20%) by the Zachary system, 11 patients (1%) by an unspecified internal grading system, 10 patients (1%) by the Haas scale, and 4 patients (1%) by the Louisiana State University Health System (LSUHS) scale (Table 3).

**Table 3.** Overall Demographics of Patients.

Category	n (%)
Total patients	751
Technique	
Nerve graft	351 (46)
Tendon transfer	339 (45)
Nerve transfer	64 (9)
Grade	
Poor	177 (24)
Neutral	154 (20)
Good	428 (57)
Level	
Wrist	171 (23)
Fingers	226 (30)
Scoring	
Bincaz	169 (23)
Haas	10 (1)
Zachary	147 (20)
MRC	413 (55)
Internal	11 (1)
LSUHS	4 (1)

*Note.* MRC = Medical Research Council; LSUHS = Louisiana State University Health System.

### Technique Comparison

Among patients undergoing tendon transfers, 82% achieved good results, 9% neutral results, and 9% poor results. For patients reconstructed with nerve grafting, 32% achieved good results, 28% neutral results, and 39% poor or no response. For those with nerve transfers, 59% achieved a good grade, 28% achieved a neutral grade, and 13% had poor or no response. These distributions were statistically significant (*P* < .001, Table 4).

**Table 4.** Analysis by Surgical Technique.

Surgery type	Good (%)	Neutral (%)	Poor (%)
Nerve graft	113 (32)	101 (28)	137 (39)
Tendon transfers	277 (82)	30 (9)	32 (9)
Nerve transfers	38 (59)	18 (28)	8 (13)

### Comparisons of Outcomes at Various Anatomical Levels

All 3 reconstructive modalities were performed to restore wrist extension. Tendon transfers had 96% good outcomes and 4% poor outcomes. Nerve grafts had 46% good outcomes and 46% neutral outcomes. Nerve transfers had 71% good, 19% neutral, and 10% poor outcomes. All 3 distributions were statistically significant (*P* < .001, Table 5).

**Table 5.** Analysis by Technique Based on Level of Function Targeted.

Surgery type	Good (%)	Neutral (%)	Poor (%)
Wrist			
Nerve graft	6 (46)	6 (46)	1 (8)
Tendon transfer	94 (96)	2 (2)	4 (4)
Nerve transfer	41 (71)	11 (19)	6 (10)
Fingers			
Nerve graft	14 (52)	5 (19)	8 (30)
Tendon transfer	113 (77)	6 (4)	27 (18)
Nerve transfer	27 (68)	18 (23)	8 (10)

Loss of finger extension was addressed with all 3 techniques. Tendon transfers resulted in 77% good, 4% neutral, and 18% poor results. Nerve grafts had 52% good, 19% neutral, and 30% poor outcomes. For nerve transfers, 68% had good, 23% had neutral, and 10% had poor outcomes. Tendon transfers were statistically superior to nerve-based reconstruction (*P* < .001), but nerve grafts and transfers were equivalent (Table 5).

As wrist and finger/thumb extension outcomes were reported for tendon transfers, nerve transfers, and nerve grafts, we studied those techniques with further subgroup analysis. Nerve transfers had superior results for wrist extension when compared with finger extension (*P* = .037); the same held true for tendon transfers (*P* = .002). Nerve grafting had improved outcomes for finger extension compared with wrist extension (*P* = .024, Table 6).

**Table 6.** Comparison by Level of Muscle Targeted Based on Technique.

Surgery location	Good (%)	Neutral (%)	Poor (%)
Nerve graft			
Wrist	6 (46)	6 (46)	1 (8)
Finger	14 (52)	5 (19)	8 (30)
Tendon transfer			
Wrist	94 (96)	2 (2)	4 (4)
Finger	113 (77)	6 (4)	27 (18)
Nerve transfer			
Wrist	41 (71)	11 (19)	6 (10)
Finger	27 (68)	18 (23)	8 (10)

### Comparisons Within Techniques by Grading System

To perform this study, we converted known grading scales into a single scoring system. To evaluate our reclassification, we compared the distribution of outcomes evaluated by each grading system with one another. We found that nerve transfer and nerve graft patients had equivalent distributions across all classifications (*P* = .232 and .08, respectively). Tendon transfers had a statistical difference between Bincaz and Zachary scoring systems (*P* < .001), explained by Bincaz scores being categorized in 2 values and Zachary in 3 (Table 7).

**Table 7.** Comparison of Outcome by Grading System Based on Technique.

Grading scale	Good (%)	Neutral (%)	Poor (%)
Nerve grafting			
MRC	106 (31)	99 (28)	135 (40)
Internal	5 (71)	0	2 (29)
LSUHS	2 (50)	2 (50)	0
Tendon transfers			
Bincaz	145 (86)	0	24 (14)
Haas	9 (90)	1 (10)	0
MRC	11 (85)	2 (15)	0
Zachary	112 (76)	27 (18)	8 (6)
Nerve transfers			
MRC	34 (57)	18 (30)	8 (13)
Internal	4 (100)	0 (0)	0 (0)

*Note*. MRC = Medical Research Council; LSUHS = Louisiana State University Health System.

We performed a comparison of tendon transfer, nerve transfer, and nerve grafting based on papers with MRC grading alone. Analysis demonstrated that all 3 surgical techniques were statistically different (*P* < .001), reflecting overall study findings and validating our results.

## Discussion

Radial nerve palsy results in loss of extension of the wrist, fingers, and thumb. Techniques developed to address these deficits include tendon transfers, nerve grafts, and nerve transfers. Tendon transfers provide dependable outcomes at any time after injury but may not provide fine motor control. In contrast, both nerve grafts and nerve transfers should theoretically provide more nuanced controlled movements but must be performed within a narrow timeframe to achieve distal muscle reinnervation. Despite clinical acceptance of all 3 techniques, there remains a paucity of data in the literature to support the superiority of one technique over the others for treatment of isolated radial nerve palsy.

Our study demonstrates that tendon transfers provide more reliable outcomes when compared with nerve-based reconstruction, as measured by rubric grading scales across heterogeneous samples. Superiority is consistent for both wrist and finger extension. These results suggest that tendon transfers should always be considered in radial nerve palsy, even if nerve-based reconstruction is an option.

Although tendon transfers require synergistic muscle function, adequate excursion, and ideal directions of pull, expendable donor muscle-tendon units are almost always available and require no reinnervation, increasing reproducibility. Nerve-based reconstruction requires nerve fibers to regenerate across 1 or 2 nerve coaptations and reinnervate target motor end plates before irreversible atrophy of the muscle occurs. This process is more likely to be successful in muscles with proximal branches off motor nerves; elbow muscles are innervated proximal to wrist muscles, which are innerved proximal to muscles affecting the digits, explaining why wrist and finger extension have better results after tendon transfers than after nerve-based reconstruction.^39^

In 1917, Robert Jones published a series of tendon transfers for radial nerve palsy, which have since been expanded and refined by other surgeons.^40^ In particular, Chuinard used the FDS transfer, Kruft described normal function after the Merle d’Aubigne procedure, and Ropars used the FCR transfer instead of the FCU transfer.^24,26,32^ This work was continued by Moussavi, Raskin, Agarwal, and Al-Qattan.^21,22,25,29^ Interestingly, all these authors used variations on the same 3 transfers: PT to ECRB/ECRL, PL to EPL, and either the FCR, FDS, or FCU to the EDC. Analysis of the different donors for digit extension revealed no significant difference in rate of outcome, suggesting that any of the 3 standard tendon transfer sets are reasonable for patients with radial nerve palsy and that surgeon preference does not affect results.

More recently, hand surgeons have explored nerve-based reconstruction. The standard nerve transfer involves the FDS to the ECRB and the FCR to PIN, as performed by Rasulić et al.^11^ Elements of this transfer were performed by Garcia-Lopez et al^17^ and Bertelli et al,^13^ while Emamhadi et al^15,16^ expanded on the indications for this transfer. As all patients had the same core nerve transfers, the actual transfer does not seem to have affected results. The main difficulty in interpreting nerve transfer studies is present in the papers by Ray and Mackinnon^19^ as well as Bertelli^14^—patients with nerve transfers often also have tendon transfers and nerve grafts. An attempt to control for by eliminating those patients from the analysis showed the power of the pooled analysis over individual studies. Pooled studies allow for better control of confounding factors that are described in the original study but not adequately addressed in the results.

Nerve grafting remains the least described modality of reconstruction. In the 1940s and 1970s, Seddon^33,34^ and Dolenc^28^ published their experiences, but details are sparse, making it difficult to quantify their degree of success. More recently, Schwaiger et al,^9^ Roganovic et al,^12^ and Shergill et al^35^ discussed adequate return of function after nerve grafting. However, across all papers, the authors did not include the level of injury, making the data difficult to interpret. The important finding was that nerve grafting can be effective, although outcomes are not as robust as tendon transfer and nerve transfer.

Several authors have directly compared outcomes between reconstructive methods. Bertelli published a comparison of 13 patients undergoing the Jones tendon transfers versus 14 patients undergoing transfer of the terminal branch of the anterior interosseous nerve to the ECRB motor branch and the FCR motor branch to the PIN, demonstrating that nerve transfers achieved better outcomes at the wrist, although finger extension was more consistent in patients undergoing tendon transfers.^14^ These findings are consistent with the concept that distally innervated muscles have less chance of recovery with nerve-based reconstruction compared with proximal muscles.^39^ Bertelli et al^13,14^ also reported success with transfer of the PT motor branch to the ECRB motor branch. However, these studies do show some patient bias; tendon transfer patients were operated on a minimum of 15 months after injury, whereas the majority undergoing nerve transfers were operated on within 6 months of injury. These represent 2 distinct patient populations in terms of potential for functional recovery and bias the study results in favor of nerve transfers. Rasulić et al^11^ described 19 patients with radial nerve injury: 7 patients underwent nerve grafting, 4 patients underwent nerve transfer, and 8 patients underwent neurolysis, with no difference in outcomes based on the reconstructive modality. Compton et al^41^ compared 106 tendon transfers with 25 nerve transfers and found no difference in wrist extension. These studies have different results from our study likely due to the smaller sample size in the Rasulic and Compton studies compared with ours. Furthermore, a pooled analysis, as in our article, allows for quantitative subgroup analysis to compare techniques at various joints, demonstrating the superiority of tendon transfers at both the wrist and the finger. This subgroup analysis could not be achieved in the individual studies. Pooling multiple studies together also erases selection bias inherent in the individual studies by bringing in patients from various sources; the heterogeneity of the population thereby mimics randomization in a patient population where randomization is difficult.

There are limitations inherent in this article. By nature, a systematic review has error within the review process; we cannot guarantee that all relevant papers were captured in our search, as older papers are often not indexed online. Error within each paper includes subjective classifications of outcomes, multiple surgeons, timing of surgery, degree of injury, and mechanism of injury. Although technique itself was relatively controlled for, exact surgical steps were not. The large populations undergoing tendon transfer result in surgical heterogeneity and variability in outcomes that could introduce error in our pooled study. Finally, low numbers of patients who underwent nerve grafting may adversely compromise subgroup analysis.

Our statistical analysis methodology also adds limitation to our study. We opted to perform a pooled analysis rather than a more traditional meta-analysis. Although pooled analyses can identify trends in data that may not exist, and are thus inferior to meta-analyses, a proper meta-analysis requires specific data, such as statistical outcomes reporting or detailed descriptions of each patient. Unfortunately, these data were not available for the entire patient population identified; limiting analysis to papers with detailed information, giving us the ability to control for variables such as institution, time from injury to date of surgery especially in the case of nerve-based surgery, specific level of surgery, and so forth, would result in a large drop off in included patients. We attempted to generally control for time from surgery by looking at method of repair, as nerve-based reconstruction is only performed within a year from injury, as well as level of injury based on level of repair. Small data sets in these subgroups would eliminate the benefit of such a study, namely, the ability to offer a large sample size for analysis.

However, the heterogeneity in the pooled patients does reasonably model the clinical spectrum of patients. There is not a standard patient who sustains a radial nerve palsy. Analysis of this large group of pooled patients may better help the average hand surgeon in deciding between different surgical options. A pooled analysis can inform what the average patient may expect, which inherently has value to a vulnerable population.

The heterogeneity of outcome grading remains another limitation. A recent review by Compton et al^41^ suggested that comparisons across techniques were not possible due to the heterogeneity in reporting outcomes. However, we feel that the Bincaz, Haas, Zachary, LSUHS scales, and the MRC grading describe essentially similar outcomes: antigravity function, gravity equivalence, and less than gravity force. Furthermore, the internal grading system provided by some authors followed a similar tripartite scheme. Therefore, these scoring systems, although superficially different, may be used for a direct comparison of techniques.

To demonstrate this, we analyzed our data based on grading systems. As expected, reconstructive outcomes did not vary by system except for the comparison between the Bincaz and Zachary scores in tendon transfers. This is likely because the Bincaz score had only 2 grades whereas the Zachary score had 3 grades. As the rates of good outcomes are similar in both scoring systems, the clinical implications are minimal. When comparing patients with only MRC grading, tendon transfers had a significantly higher rate of good outcomes. This demonstrates that our grading system translation is a legitimate method of analysis as it is similar to the pooled data.

Ideally, a randomized trial should be undertaken with patients enrolled prospectively and surgery performed by the same group of surgeons at the same timepoint after injury, matched by type, degree, and location of injury with consistent grading of outcomes. This is unlikely to be feasible given the heterogeneity and scarcity of these injuries, and the small number of surgeons willing or able to perform all 3 procedures—tendon transfers, nerve grafting, and nerve transfers.

## Conclusions

The 3 reconstructive options for isolated radial nerve palsy are tendon transfers, nerve grafts, and nerve transfers. There remains no consensus on the optimal method of treatment. However, a systematic review and pooled analysis of outcomes demonstrate that tendon transfers have more reliable and guaranteed outcomes when compared with nerve transfers and nerve grafting, both of which are less predictable. These results suggest that tendon transfers should always be offered to a patient with an isolated radial nerve palsy, even if the patient qualifies for nerve-based reconstruction.
